# Bleb-Independent Glaucoma Surgery to Activate the Uveolymphatic Route of Non-Trabecular Aqueous Humor Outflow: Short-Term Clinical and OCT Results

**DOI:** 10.3390/vision6010004

**Published:** 2022-01-12

**Authors:** Vinod Kumar, Kamal Abdulmuhsen Abu Zaalan, Andrey Igorevich Bezzabotnov, Galina Nikolaevna Dushina, Ahmad Saleh Soliman Shradqa, Zarina Shaykuliyevna Rustamova, Mikhail Aleksandrovich Frolov

**Affiliations:** 1 Department of Eye Diseases, Medical Institute, RUDN University, 6 Mikluho-Maklaya St., 117198 Moscow, Russia; 79686112302@mail.ru (K.A.A.Z.); dushina_galina@mail.ru (G.N.D.); shaykuliyevna0294@gmail.com (Z.S.R.); frolovma@rambler.ru (M.A.F.); 2 Centre of Eye Microsurgery “PRO Zrenie”, 1 Gorshina St., 141407 Khimki, Russia; magnawer@yandex.ru (A.I.B.); sh1988moscow@gmail.com (A.S.S.S.)

**Keywords:** open-angle glaucoma, collagen implant, glaucoma surgery, bleb-independent glaucoma surgery, suprachoroidal space, uveolymphatic aqueous humor outflow, glaucoma implant, conjunctival lymphatic vessels

## Abstract

The deep sclerectomy technique was modified to enhance aqueous humor (AH) outflow via the non-trabecular pathway. A pilot study was carried out to assess its safety and effectiveness. Thirty-eight patients were under observation. After superficial scleral flap (4 × 4 mm), deep scleral layers were divided into three parts by three parallel-to-limbus incisions. Deep sclerectomy without creating a window in the Descemetes’ membrane was carried out in the distal part. A collagen implant was placed under the sclera of the remaining two parts with one end in the intrascleral pool. The third proximal part was excised to expose the uvea and implant. A Nd:YAG laser trabeculotomy at the surgery site was made on postoperative days 7–10. Outcome measures were IOP change, use of hypotensive medication(s), complications, and the need for a second surgery. At six months, the mean IOP decreased from 29.1 ± 9.2 mm Hg to 14.0 ± 4.3 mm Hg (*p* = 1.4 × 10^−9^); hypotensive medication use reduced from 2.9 ± 0.9 to 0.6 ± 1.0 (*p* = 1.3 × 10^−10^); complete success was achieved in 68.4% of cases and partial success was achieved in 31.6% of cases. Intraoperative and postoperative complications were rare and manageable. The OCT of the surgery site revealed the absence of bleb in all cases. Lymphatic vessels with characteristic bicuspid valves in their lumen were detected in conjunctiva near the operation site and over it in 32 patients. IOP decrease in the proposed technique was achieved by activation of the uveolymphatic route of AH outflow.

## 1. Introduction

Glaucoma is a multifactorial neurodegenerative ocular disease. Increased intraocular pressure (IOP) is the only factor that can be affected by medication, laser, or surgery. The imbalance between AH production and outflow results in increased IOP. AH outflows from the anterior chamber (AC) mainly via two pathways: the conventional (trabecular) and the nonconventional (non-trabecular) pathways. Both pathways play an equal role in AH outflow, though there is evidence of the non-trabecular pathway carrying up to 60% of the AH outflow in young people [1].

Known surgical methods in the management of open-angle glaucoma (OAG) are focused on creating an artificial pathway for AH outflow. Depending upon their mode of action, these surgeries are divided into two groups—penetrating and non-penetrating glaucoma surgeries. A classic example of penetrating glaucoma surgery is trabeculectomy, which effectively decreases IOP for a substantial period but is accompanied by a number of serious complications such as hypotony, hypotonic maculopathy, a shallow AC, choroidal effusion, hyphema, bleb leakage, and endophthalmitis [2,3]. Deep sclerectomy (DS) is a non-penetrating surgery for glaucoma. It is safer and risks fewer complications, but it has a short-term hypotensive effect [4]. The only advantage of non-penetrating glaucoma surgeries is that they prevent complications related to a sudden decrease in IOP. Nd:YAG laser goniopuncture is considered a mandatory adjuvant procedure in order to maintain the hypotensive effect of DS [5]. Fibrosis at the operation site is the main failure reason for both types of surgery. To prevent fibrosis, use of antimetabolites has been proposed [6]. Application of antimetabolites during surgery or injections during the postoperative period increase the effectiveness of these surgeries, but their use increases the risk of the development of grave complications including bleb leakage, blebitis, and endophthalmitis [6].

The non-trabecular outflow pathway is the least investigated area. To enhance AH outflow via this pathway, the existing non penetrating DS surgical technique was modified with the aim of providing AH with easy and resistance-free access from the AC to a large surface area of uveal tissue for its resorption. A two-stage technique was developed. In early operating cases, it was observed that this technique decreased IOP significantly without formation of a filtration bleb. Absence of a filtration bleb was confirmed by optical coherence tomography (OCT). In some cases, on slit lamp examination, we also observed development of transparent fluid filled vessels in the conjunctiva adjacent to the surgery site. Their nature was confirmed as lymphatic vessels (LV) by OCT. These vessels had characteristic bicuspid valves in their lumen. A case series describing our findings was reported earlier [7]. To study the safety and effectiveness of the proposed technique in IOP reduction in OAG patients and to trace and verify the possible AH outflow pathway, a pilot study was undertaken. The results of this study are reported here.

## 2. Materials and Methods

In this clinical interventional study (non-comparative case series), a total of 50 patients (17 male patients and 33 female patients; 50 eyes; average age 76.0 ± 7.6 yrs) were operated upon. Every patient underwent glaucoma surgery by the technique described below either as a standalone procedure (13 eyes, 26%) or in combination with cataract surgery (34 eyes, 68%). In three patients (3 eyes, 6%), the glaucoma procedure was performed along with intraocular lens (IOL) replacement. One surgeon (VK) conducted all the operations between 1 March 2020 and 31 March 2021.

Patient inclusion criteria were OAG, medically uncontrolled IOP, non-compliance with prescribed hypotensive medication, informed consent provided, OAG patients with visually significant cataract, OAG patients with phakic eyes or with pseudophakia and decompensated IOP after previous glaucoma surgeries, and minimum postoperative follow-up period of 6 months.

Exclusion criteria were narrow-angle glaucoma, closed-angle glaucoma, acute attack of glaucoma, neovascular glaucoma, congenital glaucoma, and phacolytic or phacomorphic glaucoma.

Outcome measures were IOP change, use of hypotensive medication(s), complications, and the need for a second surgery.

Before surgery, a comprehensive ophthalmological examination was carried out. For visual acuity (VA) assessment, Snellen’s chart was used. The VA values were converted to logMAR for analysis purposes. IOP was measured by an iCare tonometer (ic100, Icare Finland Oy, Vantaa, Finland) [8]. The median of three consecutive measurements was taken into consideration [9]. The IOP values were adjusted for corneal thickness using the pachymeter application of spectral domain optical coherence tomography (SOCT Copernicus Revo 80, OPTOPOL Technology Sp.z.o.o., Zawiercie, Poland). Field of vision was tested on a perimeter Perigraph Perikom (Spetsmedpribor Co. Ltd., Moscow, Russia). If native lens condition allowed an OCT glaucoma analysis (RNFL, ONH morphology, DDLS, Ganglion analysis as RNFL + GCL + IP and GCL + IPL, OU, and hemisphere asymmetry) was conducted.

The ophthalmic collagen implants (MakMedi, Moscow, Russia) were made from a natural biopolymer—the connective tissue collagen material of farm animals. Highly biologically inert and hydrophilic implants are commercially available in different shapes and sizes and are permitted for use in human beings. For our purposes, we selected rectangular implants measuring 0.1 × 2 × 5–6 mm. The implants have a unique layered-cell structure with large interlayer spaces. When immersed in fluid they swell in thickness. The frontal dimensions practically remain unchanged. As per the manufacturer’s recommendations, the swollen collagen implants act as a temporary filtering zone and maintain the level of AH outflow. With time, they are slowly resorbed by tissue fluids and replaced by filtering tissue.

### 2.1. Surgical Technique

**Figure 1:**
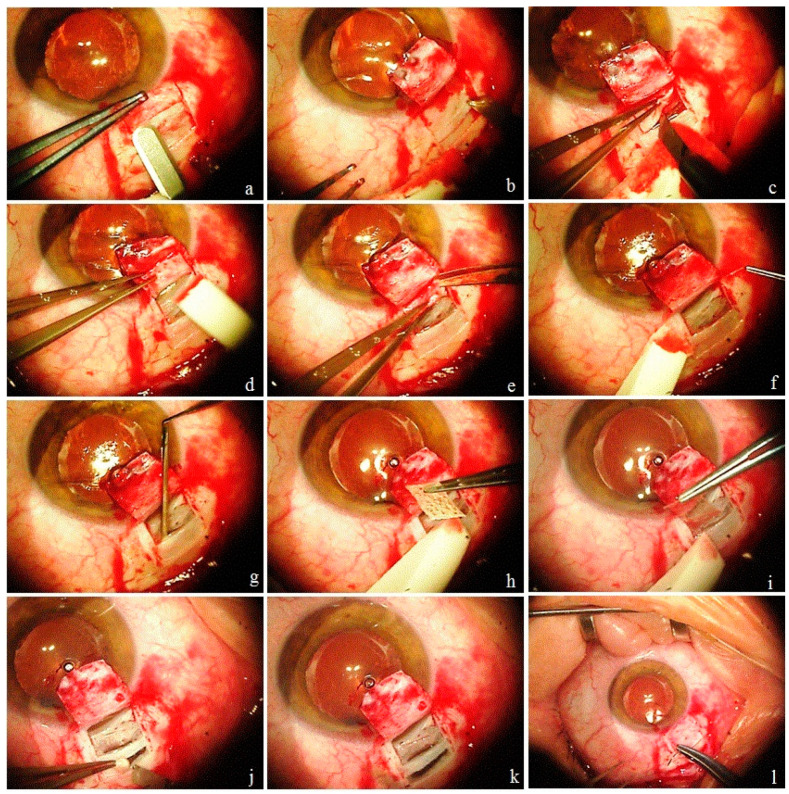
Surgical steps. (**a**) After fornix-based conjunctival peritomy, a 4 × 4 mm ½ limbal-based superficial scleral flap was fashioned. (**b**) Parallel to the limbus, and 2, 3 and 4 mm away from it, three transversal incisions up to the uveal tissue were made in the deep scleral layers to divide them into three parts. (**c**–**e**) In the distal part, a modified deep sclerectomy was performed without creation of a window in Descemete’s membrane. (**f**) A part of the juxtacanalicular connective tissue was stripped off from the inner wall of the Schlemm’s canal and removed. (**g**) Under the middle and proximal parts of the sclera, the uvea was detached with a thin blunt spatula. (**h**,**i**) A strip of collagen implant was inserted into the suprauveal space with forceps. Its anterior end lay in the intrascleral pool to act as a space maintainer. (**j**,**k**) The proximal scleral part was excised, exposing the implant and uvea. (**l**) The superficial scleral flap was replaced and fixed by two 10–0 nylon interrupted sutures placed in each corner.

At the first stage, an intrascleral reservoir (ISR) was created to receive and accumulated AH from the AC by excising the deep scleral layers. The resistance at the conventional trabecular outflow pathway was reduced by deroofing Schlemm’s canal (SC) and by removing a part of the juxtacanalicular connective tissue (JCT). To provide easy access to the suprachoroidal space (SCS), a reverse cyclodialysis tunnel (CT) was dissected under the sclera into the SCS. For long term survival of the ISR and patency of the CT, a collagen implant (CI) was implanted into it to act as a space maintainer and as a conductor for AH outflow. All surgical steps were performed without perforating the eyeball, thus, a sudden decrease in IOP was avoided. In the second stage on postoperative days 7–10, an opening was made in the TM with the help of an Nd:YAG laser. In cases with coexisting pathologies, a combined surgery was performed. First, cataract surgery (phacoemulsification with implantation of an IOL in the capsular bag) was performed, followed by glaucoma surgery. To avoid “Argentinian flag” complication in cases with an intumescent cataract, the “three step capsulorhexis” technique is preferred. In this technique, first a puncture was made in the center of the anterior capsule with a bevel down 26G disposable needle mounted on a 1 mL syringe and some liquified cortical masses were aspirated to decrease the pressure in the capsular sac. This was followed by the creation of a rhexis of smaller size. Liquified cortical masses were further aspirated from under the rhexis margins with the help of a Simcoe cannula to further decrease the pressure in the sac. The last step was to enlarge the rhexis before phacoemulsification. The surgical steps of the proposed glaucoma surgery are illustrated and explained in Figure 1 (see Supplementary Materials Video S1).

Preoperatively, the patient’s ocular hypotensive medications were not washed out. Antibacterial (sol. Levofloxacin 0.5% 1–2 drops 3 times a day) and anti-inflammatory (sol. bromfenac 0.09% once a day) medications were prescribed for a period of three days before surgery. A standard postoperative protocol was followed. Patients were evaluated the next day and based upon IOP level were instructed either to continue or discontinue hypotensive medications. Conjunctival sutures were taken out at 7 to 10 days after surgery. On this day, an Nd:YAG laser trabeculotomy was performed, following which the patient was instructed to discontinue hypotensive medications. Patients were evaluated on days 1 and 7 and then monthly up to 6 months. The first postoperative day was the day following the Nd:YAG laser trabeculotomy.

Laser trabeculotomy was performed using a slit lamp-mounted Nd:YAG laser (Optolaser, OPTOPOL Technology Sp.z.o.o., Zawiercie, Poland) and a single mirror laser gonio lens (Ocular Latina Gonio Laser Lens, Ocular instruments, Bellevue, WA, USA). Usually, 3 or 5 millijoules of energy was selected and one or more openings were created in the trabecular meshwork (TM). In a successful trabeculotomy, a pulsatile movement of AH passing through it was detected.

Postoperative assessment included VA assessment, tonometry, slit-lamp examination, ophthalmoscopy, and gonioscopy. Wherever possible, findings were documented via photography and videography. On each follow-up, the surgical site and areas adjacent to it were evaluated with the help of OCT.

**Figure 2:**
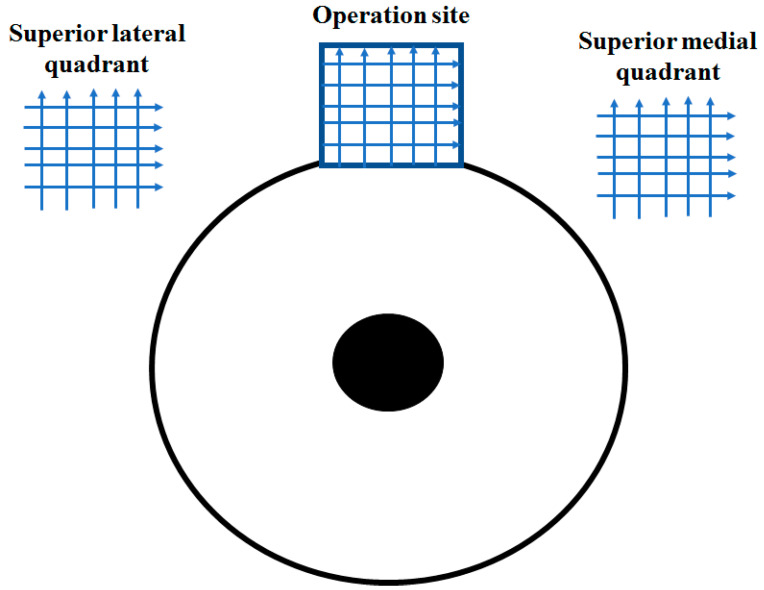
Schematic presentation of OCT evaluation of the surgery site and adjacent areas in the superior hemisphere of the eye globe. At each site, five horizontal and five vertical Raster scans were obtained.

**Figure 3:**
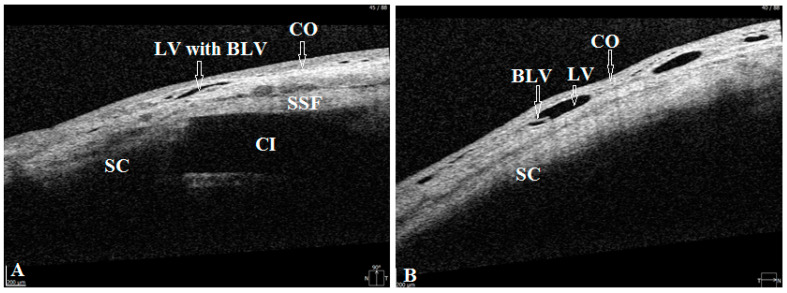
OCT evaluation of surgery and adjunct sites. (**A**) OCT scan of surgery site, showing intrascleral reservoir with collagen implant (CI), covered by superficial scleral flap (SSF) and conjunctiva (CO). No filtration bleb cavity or subepithelial microcysts are seen over the implant and in the nearby area. A lymphatic vessel (LV) with bicuspid lymphatic valves (BLV) in its lumen (white arrows) is seen running horizontally over the surgery site. (**B**) OCT scan of conjunctiva adjacent to surgery site showing a lymphatic vessel with characteristic bicuspid valve. SC = sclera.

OCT images before and after surgery were acquired by one of the trained surgeons/operators (A.I.B., A.S.S.S., G.N.D., A.J.K.A., Z.S.R.) using the commercially available SOCT Copernicus Revo 80. To perform OCT, patients were asked to look down and the upper lid was retracted to expose the surgical site, taking care to avoid pressure on the globe. Five horizontal and five vertical Raster scans 1 mm apart from each other were obtained at the operation site and adjacent to the temporal and nasal areas (Figure 2). The OCT software automatically processed the OCT images. Qualitative assessment of the scans was based on the visibility of the scleral flap, collagen implant, scleral lake, and AH drainage routes from the operation site to conjunctival vessels, microcysts, and internal fluid-filled cavities. Blebs were defined as the internal fluid-filled cavities having significantly low-reflective fluid-filled spaces adjunct to the scleral flap. The hyporeflective area was delineated by the hyperreflective conjunctiva and Tenon’s capsules in the bleb. Microcyst was defined as a small round hyporeflective space more than 10 μm in diameter in the bleb wall (in the epithelial layer) [10]. Lymphatic vessels (LV) were defined as hyporeflective spaces with characteristic bicuspid valves in their lumen (Figure 3).

To define success, the guidelines on the design and reporting of glaucoma surgical trials of the World Glaucoma Association were used [11]. Success was defined as complete if the IOP after surgery was less than the target IOP without any hypotensive medication. If additional hypotensive medication was required to achieve the target IOP, the success was considered qualified. The IOP reduction should be ≥20% and an absolute IOP between 6 mm Hg and 21 mm Hg for mild glaucomatous damage; ≥30% and IOP between 6 mm Hg and 18 mm Hg for moderate glaucoma damage, and ≥40% and IOP between 6 mm Hg and ≤15 mm Hg for advanced glaucoma damage. Failure was defined as an IOP level measured above the upper limit or below the lower limit on two consecutive visits and in the case of a need for further glaucoma intervention. In cases with preoperative medically controlled IOP with the maximum number of hypotensive medications (≥3 classes of medications), the IOP reduction was judged by percentage reduction in IOP and by reduction in the number of medications used. Fixed combination glaucoma medications were counted as two separate medications.

### 2.2. Statistics

Categorical variables were described with the frequency as a percentage. Continuous variables were described as mean with standard deviation (SD). A paired *t*-test procedure was employed to determine the significance of the mean change in IOP from the baseline to the following timepoints: 1 day, 1 week, 1 month, 3 months, and 6 months. A paired t-test was used to analyze the mean change in the number of glaucoma medications used at baseline in comparison to 1 day, 1 week, 1 month, 3 months, and 6 months. The success of treatment was expressed by a Kaplan–Meier curve. *p*-values below 0.05 were considered statistically significant. SPSS Statistics (IBM) 28.0.0.0 software for Windows 7 and Excel application of Microsoft office 365 were used for statistical processing.

## 3. Results

Out of 50 patients, 38 patients fulfilled the inclusion criteria and were included in the study. Of these, 24 patients received a combined procedure (cataract surgery and glaucoma surgery). In 12 patients, glaucoma surgery was carried out as a standalone procedure. In another two cases with IOL dislocation and high IOP, an IOL exchange along with glaucoma surgery was carried out. The demographic data of the patients and preoperative characteristics of the eyes are presented in Table 1.

### 3.1. IOP Change

**Figure 4:**
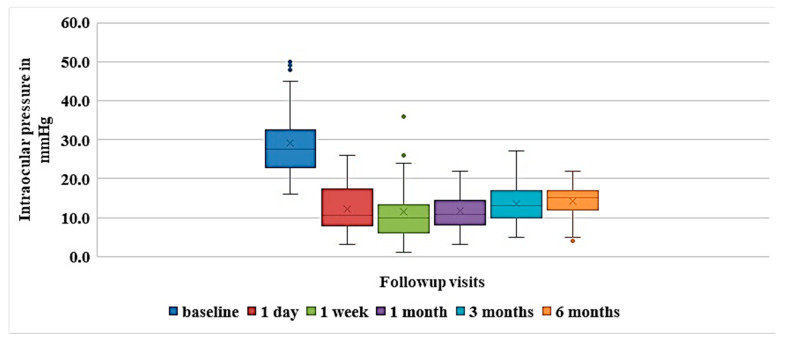
Box-and-whisker plot showing IOP change after surgery. The colored boxes represent the 25th and 75th percentile, the solid horizontal lines in the colored boxes represent the median, X represents the mean, vertical solid black lines extend the interquartile range 1.5 times, and the colored dots represent outliers.

The average postoperative observation period was 29.5 ± 7.4 weeks (95% CI 27.1–31.8). At six months, a decrease in IOP was observed in all cases. The mean baseline IOP decreased from 29.1 ± 9.2 mm Hg (95% CI 26.1–32.1) to 14.0 ± 4.3 mm Hg (95% CI 12.7–15.4) (*p* = 1.4 × 10^−9^), which constituted a decrease of >40%. IOP decrease was less than 20% in two eyes (5.3%), >20% but <30% in five eyes (13.1%), >30% but <40% in five eyes (13.1%), and >40% in 26 eyes (68.5%). In 50% of the cases (19 eyes), the IOP decrease was more than 50%. In 60.5% of cases (27 eyes), the IOP was less than 15 mm Hg. The mean IOP after surgery at different follow-up periods is presented in Table 2 and shown in Figure 4.

### 3.2. Change in Use of Hypotensive Medications

**Figure 5:**
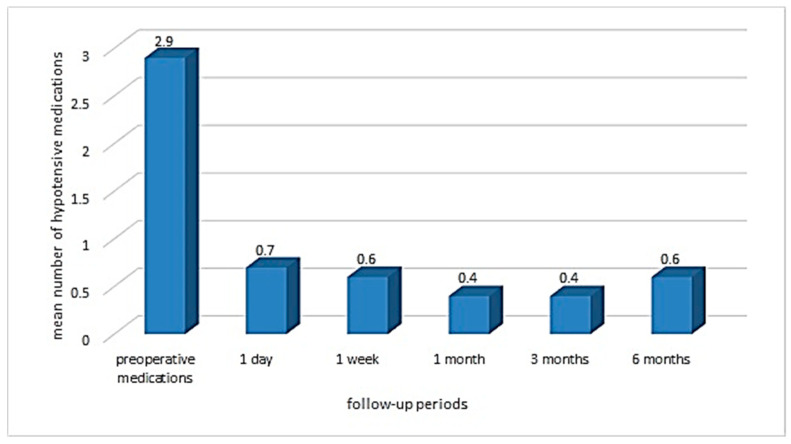
Bar diagram showing mean hypotensive medication use after surgery at different follow-up times.

At six months, the mean number of hypotensive medications used by patients reduced from 2.9 ± 0.9 (95% CI 2.6–3.2) to 0.6 ± 1 (95% CI 0.3–0.9), which constituted a reduction of 79.3% (*p* = 1.3 × 10^−10^). At all follow-up periods, more than 68% of cases were free from instillation of hypotensive drops. Changes in hypotensive medication(s) use after surgery are presented in Figure 5.

### 3.3. Visual Acuity

The average VA (logMar) before surgery was 0.5 ± 0.3. At six months, for patients who underwent the standalone procedure, the VA remained unchanged in three patients (25%), improved in five patients (41.7%), and worsened in four patients (33.3%). Deterioration in VA was mostly observed in patients with previously failed glaucoma procedures (three out of four cases). In patients with a dislocated IOL-capsule complex, VA improved in one patient and remained unchanged in the other case. In patients who underwent combined procedures, VA remained unchanged in three patients (11.5%), improved in 21 patients (80.8%), and two patients (7.7%) demonstrated worsening of VA. Deterioration of VA was related to the development of a secondary cataract.

### 3.4. Success Rate

**Figure 6:**
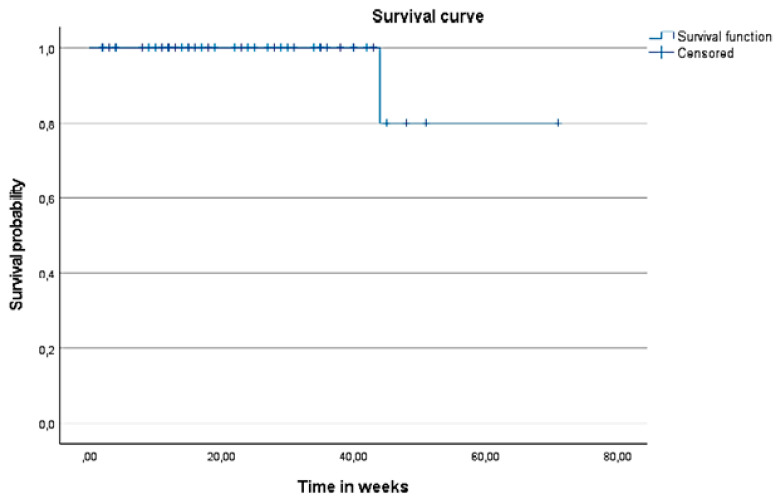
The Kaplan–Meier survival curve after surgery.

At six months, complete success was achieved in 26 cases (68.4%) and qualified success in 12 cases (31.6%). A further analysis of cases requiring additional hypotensive medication after surgery showed that two cases refused to undergo the Nd:YAG trabeculotomy, another two cases had previously failed filtration surgeries with conjunctival fibrosis at surgery site, two cases had dislocated IOLs with secondary glaucoma, one case initially had an intumescent hyper mature cataract and, in another case, the trabeculotomy opening fibrosed spontaneously, leading to increased IOP. In the other four cases, the reason could not be established. The success of treatment expressed by the Kaplan–Meier curve is presented in Figure 6.

### 3.5. Observations during Surgery

Most of the difficulties observed during surgery were related to cataract surgery for hypermature intumescent cataracts. In all nine cases with previously failed filtration surgeries, the surgeon faced certain difficulties in dissection of the conjunctival and scleral flaps. No difficulty was encountered while inserting the CI in the suprachoroidal space.

### 3.6. Observations in the Postoperative Period

During the early phase of mastering and standardizing the technique, the Nd:YAG laser trabeculotomy was performed the day after surgery, in one case leading to dehiscence of conjunctival wound requiring its resuturing in the operation theater. After this, the second step of the technique was performed 7–10 days after surgery, allowing enough time for the wound to heal. One case had spontaneous retinal hemorrhage not related to surgery, which resolved after one month with restoration of vision. Blockage of the trabeculotomy opening by iris tissue leading to increased IOP was observed in four cases; in two cases, the IOP was controlled medically, in another case, a repeat trabeculotomy near the blockage site lowered the IOP below target level, and it remained this way for the rest of the follow-up period. In one case, a second surgery was needed. Some bleeding from the SC immediately after the YAG laser trabeculotomy was observed in two cases. The hyphema resolved spontaneously within one week. There were no cases of hypotony or shallow AC during the follow-up.

### 3.7. Slit Lamp and OCT Evaluation of the Surgery and Adjacent Sites

Filtration bleb: some conjunctival swelling at the surgery site was noticed in all cases on the day following surgery. They were labelled a local tissue reaction to surgical trauma. As per the criteria mentioned above, OCT evaluation of the surgery site demonstrated absence of filtering bleb in all cases, except one, where a bleb lasting one month was identified.

**Figure 7:**
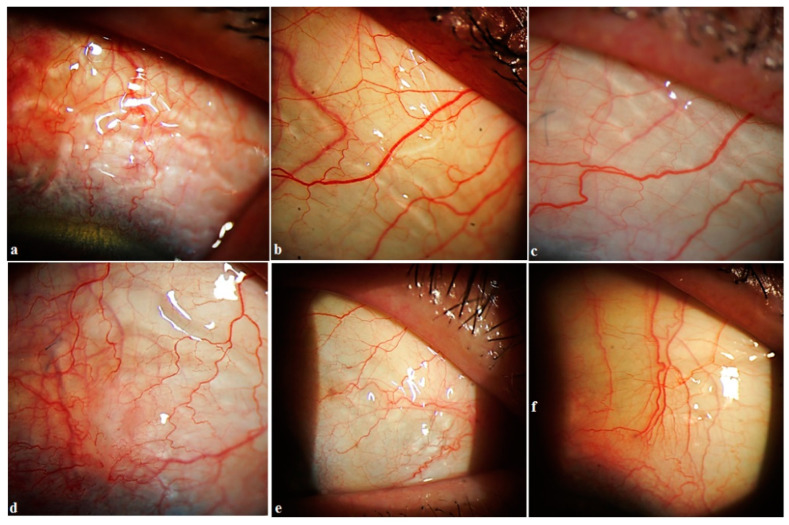
Showing different patterns of lymphatic vessels. (**a**,**b**,**d**,**e**) Lymphatics in the form of plexus. (**c**) Radially running vessels. (**f**) A case with few vessels.

**Figure 8:**
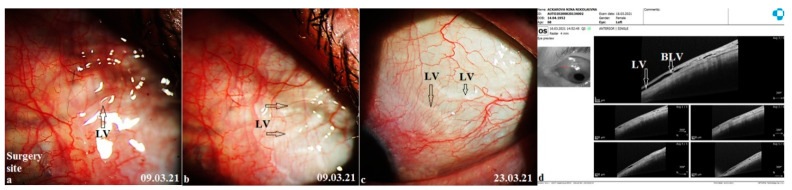
Development of lymphatic vessels immediately after the Nd:YAG laser trabeculotomy. (**a**) Showing conjunctiva medial to the surgery site immediately after trabeculotomy. Some irregular shaped swellings started appearing in the conjunctiva as aqueous humor began its exit from the anterior chamber. (**b**) Slit lamp view 15 min after trabeculotomy showing appearance of a plexus of LV. (**c**) Clinical image taken after a period of two weeks. The LV plexus is reduced in size and the IOP is below ten. (**d**) An OCT scan of the same area showing the presence of LV 1 week after the trabeculotomy. BLV = bicuspid lymphatic valves, LV = lymphatic vessels.

**Figure 9:**
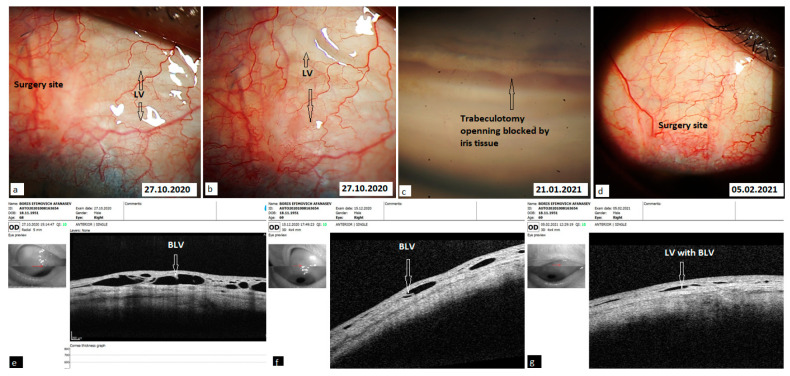
Conjunctival lymphatic plexus in the right eye of a male patient having undergone a combined procedure. An Nd:YAG laser trabeculotomy was performed seven days after surgery. (**a**,**b**) Clinical image of surgery site 2 weeks after trabeculotomy. Lymphatic vessels are filled with transparent fluid (black arrows). (**c**) After 3 months, the patient had an increase in IOP due to blockage of the trabeculotomy site by iris tissue (black arrow). A repeat trabeculotomy near to the blockage site lowered IOP below the target level and it remained this way for the rest of the follow-up period. (**d**) Image of the surgery site taken 4 months after the trabeculotomy showing absence of any bleb. (**e**–**g**) OCT scans identifying the lymphatic nature of vessels in the conjunctiva lying over the scleral flap and in the conjunctiva adjacent to the medial and lateral borders of the surgery site (white arrows). BLV = bicuspid lymphatic valves, IOP = intraocular pressure, LV = lymphatic vessels, OCT = optical coherence tomography.

Lymphatic vessels: some fluid filled transparent vessels in the bulbar conjunctiva following surgery were observed in 15 cases on slit lamp biomicroscopy. These vessels began from the surgery site, mostly on the medial side. Rarely were they identified on the temporal side. OCT revealed their nature to be LV with characteristic bicuspid valves in their lumen. No pattern in their appearance was identified (Figure 7). In some cases, they appeared immediately after the Nd:YAG trabeculotomy, especially in cases with higher IOP (Figure 8). In other cases, it took 1–2 weeks for them to appear. Their number and size were related to the IOP level. With normalization of IOP, they reduced in size and number. Clinical images and OCT scans of some of these cases are represented in Figure 8 and Figure 9.

OCT evaluation: at six months, OCT of the surgery and adjacent sites was carried out in 35 patients. Three patients refused to undergo examination. No bleb was detected in any of the cases. LV were detected in 32 of the investigated cases (91.4%). They were detected in the conjunctiva overlying the surgery site in 23 cases, in conjunctiva medial to the surgery site in seven cases, in conjunctiva lateral to the surgery site in 13 eyes, and on both sides in 12 cases. It was observed that the abundance of LV lasted from 1 to 2 months. In some cases, they remained identifiable up to 10 months. Once compensation of IOP was achieved, their number reduced considerably, and their presence became negligible.

## 4. Discussion

In the trabecular outflow pathway, AH flows through the TM into the SC and then into a network of collector channels. The TM, SC, collector channels, and distal outflow pathways function as a sophisticated organ system that works in unison to control trabecular outflow. Trabecular outflow involves a “biomechanical pump” that is powered by the ocular pulse pressure, blinking, and saccadic eye movements. The flexible TM distends and recoils in synchrony with the cardiac cycle, actively moving aqueous fluid into the SC [12].

The percentage of aqueous outflow draining via the non-trabecular pathway in humans comprises approximately 50% of outflow in young healthy individuals, but this flow declines in glaucoma [1]. In the non-trabecular outflow, AH passes through the anterior face of the ciliary muscle to reach the supraciliary and suprachoroidal spaces. From there, AH can drain via three possible routes: (1) through the connective tissue of the sclera (uveoscleral flow); (2) into the choroidal vessels and then the vortex veins (uveovortex flow); (3) into the lymphatic vessels within the ciliary body (uveolymphatic flow) [1,13,14]. Non-trabecular outflow is controlled by the resistance imparted by the muscle bundles and connective tissue of the ciliary body. Uveoscleral flow depends upon scleral permeability, which in turn depends upon IOP level. At higher IOP levels, scleral permeability is reduced. The mechanism for this increased resistance is thought to be tissue compression, reducing the spaces between collagen fibers and extracellular matrix molecules [1]. AH reaching the SCS enters the choroidal vasculature primarily by osmosis, to drain via the uveovortex pathway. The outflow is driven by a large colloidal osmotic gradient. Yucel et al. [13,14] described and proved the presence of the uveolymphatic pathway for AH drainage. The authors confirmed the presence of lymphatic endothelium in the human ciliary body.

In nature, the AH flows in a closed system with a bulk flow mechanism. After penetrating and non-penetrating glaucoma surgeries, the AH is received in the subconjunctival bleb. From there, it filters through conjunctival vessels and lymphatics. In the literature, there are few studies in which the role of conjunctival and lymphatic vessels in AH drainage from the bleb is studied [15,16,17]. Benedikt [15,16] was one of the pioneers who investigated AH drainage from a bleb using fluorescence photography after intracameral injection of fluorescein. The author concluded that AH drainage from the bleb occurs by the transconjunctival route, diffuse resorption through degenerated veins, bulk flow through lymphatic vessels and atypical aqueous veins, and outflow through normal aqueous veins. The author also observed that a filtering bleb develops if the transconjunctival route and the diffuse resorption are predominant. Newly incorporated veins and lymphatic vessels enable the drainage of the AH without a visible bleb. The author emphasized that the most important cause of the formation of these vessels is the surgical technique and recommended covering the fistula with a scleral flap, which is also necessary to obtain a physiological IOP immediately after the operation. Per his observations, if the tension is too low at the surgery site, in most cases, a filtering bleb will form. If the IOP is at a physiological level (i.e., above the episcleral vein pressure), new vessels can develop and drain the AH by bulk flow from the scleral fistula, which means there will be a good pressure-regulating effect without a filtering bleb. Using fluorescent lymphangiography, Alekseev et al. studied lymph microcirculation in the bulbar conjunctiva at a surgery site and conjunctival lymph vessels in patients with primary glaucoma before and after trabeculectomy [17]. They observed that postoperative changes in the pattern and intensity of these vessels reflected the activity of the cicatrization process at the surgery site and were closely related to the long-term hypotensive effect. A moderate narrowing of the lymph vessels was observed in cases with complete IOP compensation without additional hypotensive medication. This effect was compensated for by an increase in the quantity and volume of lymph flow. We also observed a significant decrease in all the lymph flow parameters when the hypotensive effect of surgery was insufficient. In long-term observations, a regular decrease in lymph flow was recorded. The authors suspect that this decrease could predict the deterioration of the hydrodynamic parameters. Hence, the function and distribution of conjunctival and lymphatic vessels play an important role in the regulation of AH drainage from the bleb.

The use of perioperative and postoperative antimetabolites, a common practice among glaucoma surgeons, causes a reduction in conjunctival and lymphatic vessels in the bleb and surrounding tissue, which may hamper the immunologic response against microbial flora on the conjunctival surface and may play a key role in immunopathologic mechanisms resulting in blebitis [18].

With the proposed technique, we initially expected formation of a bleb at the surgery site. Clinical observations and OCT evaluation of the surgery site indicated AH outflow via conjunctival lymphatics without the formation of a bleb. In some cases, these vessels were visible on slit lamp. The time of their appearance, along with their duration, number, shape, and sizes, differed from patient to patient. No pattern was identified in their development. In some cases, they appeared immediately after the Nd:YAG trabeculotomy, especially in cases with a higher IOP. After the trabeculotomy, some swelling was noticed at the surgery site in all cases. After a period, usually 5–10 min after the trabeculotomy, in some cases they became engorged and became visible at the scleral flap sides transforming into a plexus of transparent vessels. OCT indicated their nature to be lymphatic with characteristic bicuspid valves in their lumen. Most probably they were the preexisting conjunctival lymphatic vessels to which the AH finds its way. With the excessive AH, they swelled. These observations are identical to observations sometimes noticed while injecting anesthetics subconjunctivally, when the anesthetic finds its way to conjunctival lymphatics, and they become visible. With the onset of normal AH outflow via these vessels, the LV became less prominent and reduced in size and number. This has been clearly demonstrated in the case reported in Figure 8. In other cases, it took some time for them to develop, usually 2–3 weeks. With normalization of IOP, they reduced in size and number.

Clinically, in most of the cases, the LV were visible on the medial side of the superior hemisphere of the globe. This can be explained by the organogenesis and distribution of the ocular lymphatic vessels in the anterior eye [19]. The limbal and conjunctival lymphatic networks are progressively formed from a primary lymphatic vessel that grows from the nasal-side medial canthus region at birth. This primary lymphatic vessel branches out to invade the limbus and conjunctiva and bidirectionally encircles the cornea. As a result, the distribution of the ocular lymphatics is more pronounced at the nasal side and the limbal lymphatics are directly connected to the conjunctival lymphatics. New lymphatic sprouts are produced mainly from the nasal-side limbal lymphatics, making the nasal side of the eye more responsive to fluid drainage. This is what we observed in our study as well.

**Figure 10:**
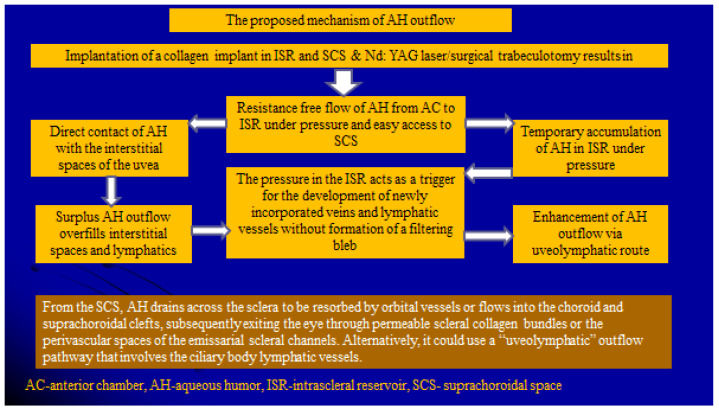
The proposed mechanism of aqueous humor outflow after the proposed glaucoma surgery.

The mechanism of AH outflow by the proposed technique may be explained as follows. AH bypasses resistance at the TM via the trabeculotomy opening and travels as bulk flow from the AC to the ISR, where it accumulates temporarily and creates a higher pressure than that in the episcleral veins. In the ISR, it comes into contact with the interstitial spaces of the uvea from where the uveolymphatic outflow pathway begins. Surplus AH outflow overfills the interstitial spaces and lymphatics. The pressure in the intrascleral space acts as a trigger for the development of newly incorporated veins and lymphatic vessels without the formation of a filtering bleb. When a balance is reached between AH inflow from AC to ISR and AH outflow via the uveolymphatics route, the clinically visible lymphatic vessels become less prominent and reduced in size and number. The proposed mechanism is illustrated in Figure 10.

Intrascleral implantation of biocompatible collagen implants is recommended to enhance the success rate of DS and to lessen the effect of fibrosis at the surgery site [20,21,22]. These implants maintain the space in the scleral bed, provide a support for the elimination route of aqueous humor and act like a sponge, carrying the liquid by capillary action. The placement of the CI in the way described in this technique plays an important role in the activation of the uveolymphatic outflow pathway. This is indirectly supported by our observations in cases where a classical DS was performed (with the creation of a trabeculo-Descemet’s window without a laser trabeculotomy) with placement of a CI as described above, where LV were observed on slit lamp examinations with a significant decrease in IOP. Mitwally et al. [23] also demonstrated a greater IOP-lowering effect of DS with suprachoroidal CI than with DS and intrascleral implantation. The role of the implantation of a non-absorbable collagen implant (Xenoplast, Dubna-Biofarm, Moscow, Russia) in the SCS in decreasing IOP has been studied by Shradka A.S. et al. [24] and Kumar V. et al. [25]. As per these authors, Xenoplast may be implanted either by an ab externo or ab interno approach. The main drawback is its coarse surface, which makes implantation traumatic to uveal tissue, leading to fibrosis and blockage of the cyclodialysis tunnel. The implant used in this study is thin and stiff in its dry state with smooth surfaces. Its implantation is easy and atraumatic. Notably, in the proposed technique, the exposure of uveal tissue surface for AH resorption was maximized by additionally incising a strip of deep scleral layers posterior to the scleral bridge (Figure 1j,k).

The proposed technique has certain advantages. All surgical steps are performed without perforating the eyeball; hence, all complications related to the sudden decrease in IOP are either minimized or completely avoided. In the proposed technique, the enhanced aqueous outflow occurs via the uveolymphatic route, which is a natural outflow pathway. This outflow does not depend upon a filtration bleb, hence there is no need for the use of antimetabolites to counter fibrosis at the surgery site.

This technique has certain drawbacks as well. First, it is an ab externo procedure and requires lot of tissue dissection, resulting in surgical trauma and prolongation of surgery time. Second, it is a two-stage surgery and needs close monitoring of patients in the early post-operative period. Third, there is a possibility of blockage of the trabeculotomy opening by iris tissue, resulting in an increase in IOP. A pre-, peri- or postoperative peripheral iridotomy, adjacent to the area of proposed intervention, may help to avoid this complication.

A shortcoming of this study is that this a small case series with short term follow-up. Randomized, controlled, and comparative studies with longer follow-up and larger groups are required in order to confirm the efficacy and safety of this technique.

## 5. Conclusions

The results of this pilot study enable us to conclude that bleb-independent glaucoma surgery is safe and effective at decreasing IOP and hypotensive medication use in glaucoma patients. IOP decrease by the proposed technique is achieved by activation of the uveolymphatic route of the non-trabecular outflow pathway without formation of a filtering bleb. For predictable hypotensive outcomes, every caution should be taken in glaucoma surgery to preserve the lymphatics.

## 6. Patents

1. Application for grant a patent of Russian Federation (dated 31 March 2021, registration number—2021108788).
2. Application for grant a patent of Russian Federation (dated 20 August 2021 registration number—2021124796).

## Figures and Tables

**Table 1:** Demographic data of patients and preoperative characteristics of the eyes.

	Number of Patients (%) Mean ± SD [95% CI]
Sex: male/female	13/25
Average age	75.7 ± 7.6 [73.3–78.2] yrs
Minimum/maximum age	59/87 yrs
Eye: right/left	17/21
Glaucoma type:	
Primary open angle	27
Refractory	9
Secondary glaucoma	2
Severity of glaucoma: moderate/severe	14/24
Baseline IOP in mm Hg	29.1 ± 9.2 [26.1–32.0]
Mean number of hypotensive medications used before surgery	2.9 ± 0.9 [2.6–3.2]
1 type of hypotensive medication	1
2 types of hypotensive medications	16
3 types of hypotensive medications	10
4 types of hypotensive medications	10
5 types of hypotensive medications	1
Number of patients having undergone previously glaucoma surgeries	9
Glaucoma surgery performed 1 time	7
Glaucoma surgery performed 2 times	1
Glaucoma surgery performed 3 times	0
Glaucoma surgery performed 4 times	1
Previously performed glaucoma surgery (number of eyes)	
Trabeculectomy	2
Segmental dilation of Schlemm’s canal	8
Laser iridotomy	1
Cyclodialysis ab externo with implantation of collagen implant (Xenoplast) in CT	2
Lens condition	
Cataract (hypermature intumescent cataract-2 eyes; severe phacodonesis-3 eyes)	24
Pseudophakia (2 cases with IOL dislocation)	14
Comorbidities	
Macular degeneration (1 case had 3 intravitreal injections of antiVEGF; epiretinal fibrosis-1 eye	4
Pseudoexfoliative syndrome	19
High myopia	3
Diabetes mellitus	3

where CI = confidence interval; CT = cyclodialysis tunnel; IOL = intraocular lens; IOP = intraocular pressure; and SD = standard deviation.

**Table 2:** Intraocular pressure changes after surgery.

Follow-Up Period	IOP (mm Hg) Mean ± SD [95% CI]	IOP (mm Hg) Reduction Mean ± SD [95% CI]	% Reduction in IOP Mean ± SD [95% CI]	*p* Values
Baseline	29.1 ± 9.2 [26.1–32.1]	-	-	-
1 day	12.2 ± 6.4 [10.2–14.3]	16.8 ± 11.4 [13.2–20.4]	53.6 ± 27.8 [44.7–62.4]	5.0 × 10^−11^
1 week	11.4 ± 7.5 [9.0–13.8]	17.7 ± 12.2 [13.8–21.5]	56.7 ± 32.2 [46.5–66.9]	9.0 × 10^−11^
1 month	11.6 ± 4.4 [10.2–13.1]	17.4 ± 8.6 [14.7–20.1]	57.8 ± 17.6 [52.2–63.4]	7.4 × 10^−15^
3 months	13.6 ± 4.5 [12.2–15.0]	15.5 ± 9.5 [12.5–18.5]	50.2 ± 17.7 [44.6–55.9]	3.7 × 10^−12^
6 months	14.0 ± 4.3 [13.3–16.1]	15.0 ± 9.2 [12.1–18.0]	48.4 ± 18.6 [42.5–54.3]	3.5 × 10^−12^

where CI = confidence interval; IOP = intraocular pressure; SD = standard deviation.

## Data Availability

The data presented in this study are available on request from the corresponding author. The data are not publicly available due to case histories of patients.

## Supplementary Materials

The following are available online at https://www.mdpi.com/article/10.3390/vision6010004/s1, Video S1: Bleb-independent glaucoma surgery.
